# Application of Machine Learning to Predict Dielectric Properties of In Vivo Biological Tissue

**DOI:** 10.3390/s21206935

**Published:** 2021-10-19

**Authors:** Branislav Gerazov, Daphne Anne Caligari Conti, Laura Farina, Lourdes Farrugia, Charles V. Sammut, Pierre Schembri Wismayer, Raquel C. Conceição

**Affiliations:** 1 Faculty of Electrical Engineering and Information Technologies, Ss Cyril and Methodius University in Skopje, 1000 Skopje, North Macedonia; gerazov@feit.ukim.edu.mk; 2 Department of Physics, University of Malta, MSD 2080 Msida, Malta; dcaligariconti@gmail.com (D.A.C.C.); lourdes.farrugia@um.edu.mt (L.F.); charles.v.sammut@um.edu.mt (C.V.S.); 3 Translational Medical Device Lab, National University of Ireland Galway, H91 TK33 Galway, Ireland; laura.farina@nuigalway.ie; 4 Department of Anatomy, University of Malta, MSD 2080 Msida, Malta; pierre.schembri-wismayer@um.edu.mt; 5 Instituto de Biofísica e Engenharia Biomédica, Faculdade de Ciências da Universidade de Lisboa, 1749-016 Lisboa, Portugal

**Keywords:** ex vivo and in vivo dielectric properties, tissue hydration, machine learning modelling

## Abstract

In this paper we revisited a database with measurements of the dielectric properties of rat muscles. Measurements were performed both in vivo and ex vivo; the latter were performed in tissues with varying levels of hydration. Dielectric property measurements were performed with an open-ended coaxial probe between the frequencies of 500 MHz and 50 GHz at a room temperature of 25 °C. In vivo dielectric properties are more valuable for creating realistic electromagnetic models of biological tissue, but these are more difficult to measure and scarcer in the literature. In this paper, we used machine learning models to predict the in vivo dielectric properties of rat muscle from ex vivo dielectric property measurements for varying levels of hydration. We observed promising results that suggest that our model can make a fair estimation of in vivo properties from ex vivo properties.

## 1. Introduction

A significant body of fundamental research has been conducted by researchers to measure the dielectric properties of biological tissue (e.g., [1,2,3]). Such studies are of extreme importance to the correct modelling of the electromagnetic behaviour of biological systems, which can be used in a number of applications. Traditionally, the dielectric properties of biological tissues have been studied to address requirements defined by dosimetry studies and often this determined the methodology used. Recently, there has been a great interest in accurate knowledge of the dielectric properties by the microwave medical community [4,5,6], as these facilitate the correct design and development of medical electromagnetic imaging devices [7,8].

Many historical studies have reported dielectric data obtained using different protocols for measuring the dielectric properties of biological tissues. Many studies reported data measured under specified different conditions—such as temperature, frequency or time from excision—(or have failed to report the conditions of experiments) and have also reported different techniques for measuring dielectric properties such as coaxial probe, transmission line, free space, resonant cavity, parallel plate and inductance measurement method [9].

An international networking project, COST Action (MyWAVE, CA17115), has begun with the objective of reviewing current protocols for measuring biological dielectric and thermal properties and converging toward a standard protocol that will allow for data collected across sites to be comparable, eventually merging into a single database. One of the groups involved in this international project has proposed the use of the “Minimum Information Model for Dielectric Measurements of Biological Tissues (MINDER)”, which provides “a framework for recording and storing dielectric data and metadata for measurements conducted on biological tissues”. By ensuring that common metadata is collected across sites, then it is ensured that “the generation of data is repeatable, interoperable, and re-usable” [10,11].

One recurrent discussion within this research community is how to correlate ex vivo with in vivo dielectric properties. Ex vivo properties are easier to measure, whereas in vivo properties are preferred to create realistic electromagnetic models of biological tissue. Additionally, several works in the literature dealt with measurements of tissue dielectric properties in ex vivo conditions; nevertheless, it is important to investigate whether the irreversible variations, occurring in biological tissues after their excision from the host, affect the dielectric properties. Ex vivo measurements on specimens from surgical resections are subject to confounding factors and depend on the frequency range under investigation. At microwave frequencies, the dielectric response is often referred to as the γ-dispersion, which is mainly due to the interaction with water content [12]. Therefore, the variability of hydration in the sample under test significantly influences the resulting measured dielectric properties. It has been shown that in vivo properties can be estimated by ex vivo measurements given that the variation in hydration between the two samples does not exceed 10% [13,14]. Many studies on dielectric properties emphasise the importance of tissue hydration, but very few studies quantify these variations [13,15]. One of the first studies that quantified water content goes back to 1992 by Campbell and Land [16], reporting no sufficient data for comparison. Moreover, they used a resonant technique that requires extensive sample preparation which can compromise the accuracy of the water content evaluated in the sample under test.

Dielectric properties are easier to measure ex vivo, but the in vivo properties represent a more realistic scenario with more accurate measurements of the electromagnetic properties of the biological tissue, as required for the design of microwave medical devices and applications. Thus, it is key to understanding the correlation between the ex vivo and in vivo properties in order to use the former to predict the latter. In this paper, we show preliminary results that demonstrate which information from the recorded metadata can help predict and infer the in vivo dielectric properties from available ex vivo dielectric properties.

## 2. Materials

This work investigated the ex vivo dielectric properties of biological tissue samples (i.e., rat muscle) and to studied how the variation in tissue hydration may help predict in vivo dielectric properties.

Spectroscopy measurements were conducted using an Agilent Slim Form 85,070 probe connected to a R&S ZVA-50 Vector Network Analyzer (VNA) in a frequency range between 500 MHz and 50 GHz in a linear scale. The VNA measured the complex reflection coefficient, S_11_, which was converted to complex permittivity, ε*, using the Agilent software 85070E (Agilent, 2012).

Each tissue sample underwent a dehydration process using a loss-on drying method. The dielectric properties of each tissue sample were measured in vivo in situ, when freshly excised and after dehydration, and then correlated with the percentage loss of water content. Various techniques were studied to determine the water content of the samples: loss-on-drying (LOD) method, centrifugation and freezing, with LOD being the most reliable method for biological tissues and, thus, used for the purpose of this study. In the meantime, intermediate measurements were made, obtaining a set of dielectric data and their corresponding water content. Levels of dehydration between 10% and 70% were achieved. The dielectric measurement data of biological tissues in vivo and ex vivo used in this study are reported in [13] which outline the experimental protocol adopted for the measurement collection. We would like to emphasise that the in vivo and ex vivo samples from each animal were the same.

## 3. Data Processing and Discussion of Results

We modelled the ex vivo dielectric data in several machine learning models. The database comprised a large number of observations (each measurement of complex permittivity) which were characterised by a number of input features, for example, the frequencies at which the measurements were recorded and the level of hydration for a tissue. We knew the ground truth of the in vivo measurement of complex permittivity for each ex vivo observation. We used a nested cross-validation methodology to train and test our model. In the outer cross-validation loop, we took each rat as a test set and conducted an inner cross-validation loop with the rest of the rats. In the inner loop, we performed a randomised search of the model’s hyperparameters using a leave-one-out group (rat) cross-validation. The best performing hyperparameters were then used to train a model in the whole inner loop set, which was then evaluated on the test set in the outer loop. Finally, the score for each model was calculated as an average score across the outer loop iterations. We present the regression calculation of the in vivo complex permittivity of the validation set and compare these with the ground truth measured results.

We completed the following five tasks:

Task 1—load the measurement data set in Python;
Task 2—explore and visualise the data;
Task 3—structure the data for regression model training and evaluation;
Task 4—choose a set of machine learning regression models to evaluate;
Task 5—train and evaluate the models with the data in a nested cross validation loop tuning their hyperparameters.

The data set comprised measurements of the relative permittivity $\varepsilon_{r}$ of rat muscle tissue given by:

(1) $$\varepsilon_{r}=\frac{\varepsilon }{\varepsilon_{0}},$$

(2) $$\varepsilon_{r}=\varepsilon_{r}^{\prime }-i\varepsilon_{r}^{\prime \prime },\;$$

where ε* is the actual permittivity of the tissue, and *ε*_0_ is the permittivity of vacuum. $\varepsilon_{r}^{\prime }$ and $\varepsilon_{r}^{\prime \prime }$ are the real and imaginary parts of the relative permittivity, respectively. Relative permittivity was measured in the range of 101 frequency data points from 500 MHz to 50 GHz, with a step of 495 MHz (101 frequency measurements). Both in vivo and ex vivo properties were measured.

For ex vivo properties, permittivity was tracked across several stages of dehydration which were thermally induced and determined by the percentage of weight loss. Four rats were used for multiple measurements, with the ex vivo dehydration levels varying between the rats. For each rat, two thigh muscles were measured in a varying number of randomly chosen locations to obtain a good estimate of the average permittivity of the tissue. In total, 3535 in vivo and 33,229 ex vivo data points at different frequencies, for a total of eight muscle samples, at every dehydration state (in ex vivo), were available in the data set. In the data set we used for this analysis, we discarded some of the data due to the noted calibration problems with the measurement device. The final spread of the data used across the different rats is given in Table 1. The total number of measured data points used was 25,149, taken from 249 measurements (each at 101 frequencies).

### 3.1. Exploratory Data Analysis

#### 3.1.1. Data Loading, Cleaning and Restructuring

In the first step of our exploration, we loaded the measured data points, originally available across a number of Excel spreadsheets into Pandas (Python Data Analysis Library https://pandas.pydata.org/, accessed on 23 September 2021) dataframes. To do so, we first re-organised the column labels in the spreadsheets. We then loaded the data into Python and eliminated the measurements with known calibration issues. Finally, we restructured all the data into a single master dataframe containing one data point per row, i.e., 25,149 rows.

#### 3.1.2. Number of Measurements and Dehydration Levels

**Figure 1:**
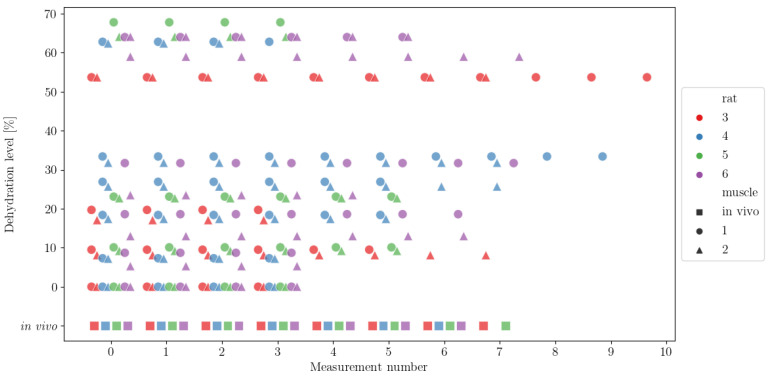
Spread of dehydration levels and number of measurements per level per rat. The in vivo measurements are shown below the ex vivo measurements. The colour codes for the four rats are red, blue, green and purple for rats labelled 3, 4, 5 and 6, respectively. In vivo muscle measurements are represented in coloured squares (there is no muscle designator for the in vivo measurements), whereas ex vivo measurements of measured muscles 1 and 2 are represented in circles and triangles, respectively. Scatter points were plotted with an added offset to avoid their overlap.

To visualise the spread of the dehydration levels and number of measurements per rat/level, we plotted the scatter plot shown in Figure 1. The dehydration level shown below 0% encodes in vivo measurements. The colour codes for the four rats are red for rat 3, blue for rat 4, green for rat 5 and purple for rat 6. In vivo muscle measurements are represented in coloured squares (there is no muscle designator for the in vivo measurements), whereas ex vivo measurements of the measured muscles 1 and 2 are represented in circles and triangles, respectively. Scatter points were plotted with an added offset to avoid their overlap. We can observe that the number of measurements went from four measurements for each rat–muscle combination of ex vivo without dehydration, up to 11 measurements for rat 3 muscle 1. The average number of measurements per rat–muscle combination was 5.8. We can further observe that the two muscles of rat 3 were measured at three dehydration levels, as were those for rat 5; the muscles for rat 4 were measured at five dehydration levels, and the muscles for rat 6 were measured at eight dehydration levels.

#### 3.1.3. Visualising the Measurements Per Frequencies

**Figure 2:**
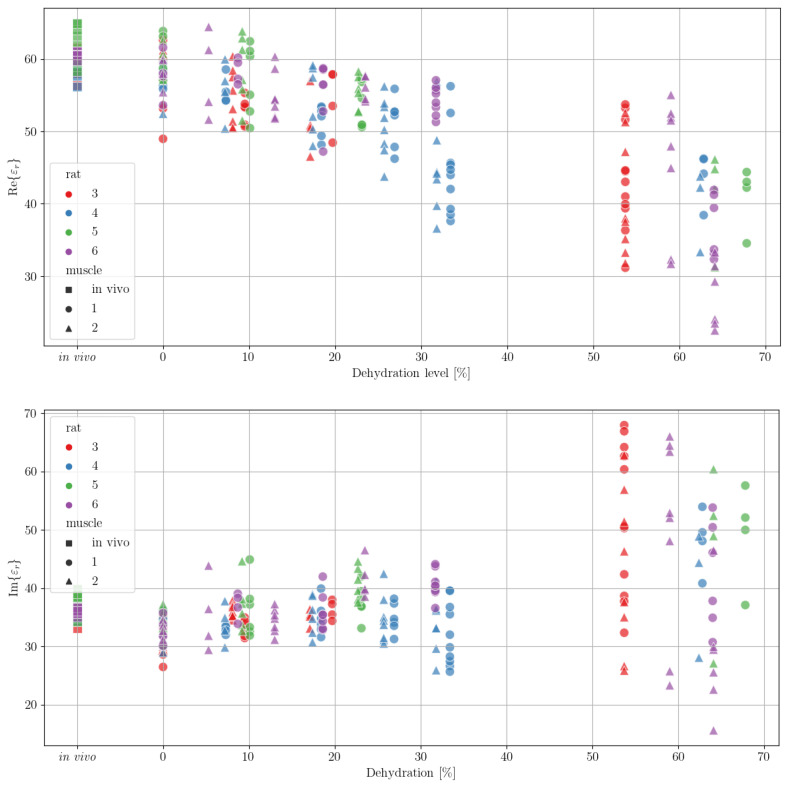
Measurements of the real (**top**) and imaginary (**bottom**) part of the permittivity (*ε*′ and *ε*″, respectively) for a frequency of 500 MHz. Measurements were performed on 4 rats (labelled as 3, 4, 5 and 6, colours red, blue, green and purple, respectively); in vivo measurements were made on a single muscle (square), and ex vivo measurements were performed on two muscles (1 and 2, circle and triangle shapes, respectively).

To visualise the measurements, we plotted the real and imaginary part of the *ε_r_* ($\varepsilon_{r}^{\prime }$ and $\varepsilon_{r}^{\prime \prime }$, respectively) for a set frequency as shown in Figure 2. We can observe that the data did capture the general dynamics of permittivity change for increasing dehydration levels. We can also observe that the variability in the measurements also increased with the increase in dehydration. Moreover, we can note that the inter-rat spread of the measurements was rather consistent, although there were some inconsistencies, e.g., measurements at the ~30% dehydration level were only recorded for rats 4 and 6. Finally, we can note that there were no measurements made between the levels of 40% and 50% dehydration for any of the rats. One can also infer the technical challenges behind ensuring specific dehydration levels at higher percentages, as it was not possible to measure at the same dehydration level: ~54% was the highest dehydration possible in rat 3, ~59% and 64% in rat 6, ~62% in rat 4 and ~64% and ~68% in rat 5. In addition, the measurements at higher dehydration levels varied more compared to measurements at lower dehydration levels.

#### 3.1.4. Visualising the Measurements for all Frequencies in 3D

**Figure 3:**
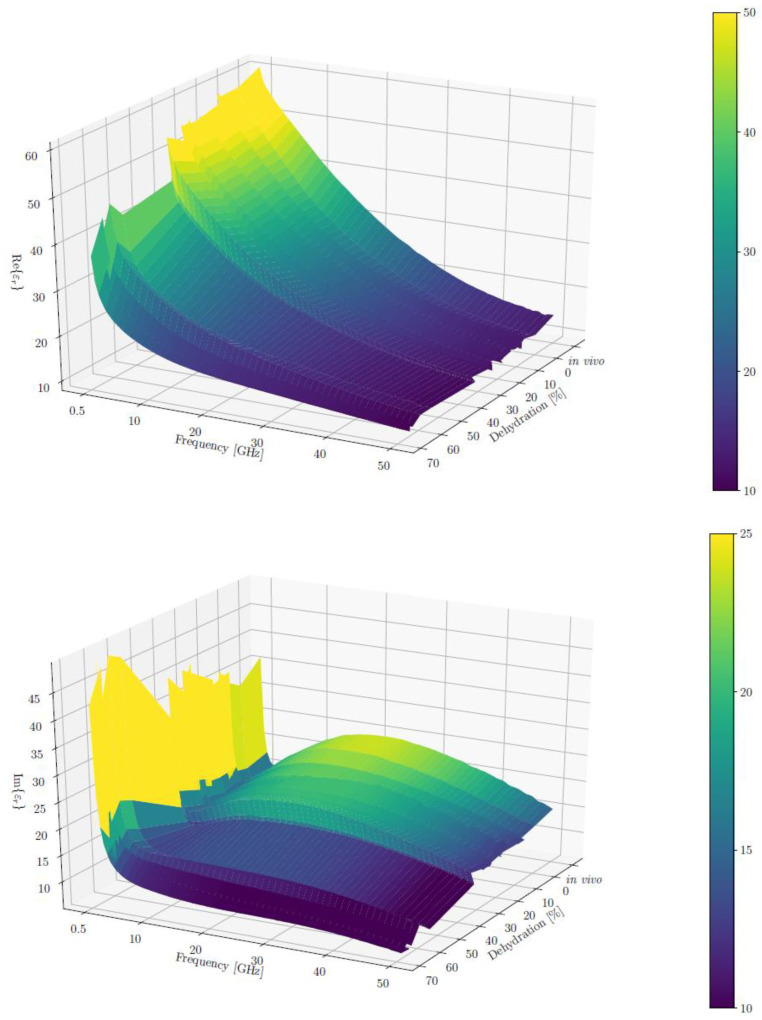
Average real (**top**) and imaginary (**bottom**) parts of the relative permittivity ($\varepsilon_{r}^{\prime }$ and $\varepsilon_{r}^{\prime \prime }$, respectively) across all frequencies in 3D. Note that the colour bar limits were adjusted for better contrast; the actual ranges are 7–65 for Re($\varepsilon_{r}$) and 3–68 for Im($\varepsilon_{r}$).

To plot $\varepsilon_{r}^{\prime }$ and $\varepsilon_{r}^{\prime \prime }$ across all frequencies using a 3D plot, we calculated the mean and standard deviation of both real and imaginary parts, respectively, for each dehydration level, grouping the measurements from different rats and muscles. The resulting averages of *ε*′ and *ε*″ are shown in the two surface plots in Figure 3. We can observe that ε’ (the real part) had a more monotonous change with respect to both the frequency and dehydration levels. It decreased steadily for higher frequency and dehydration levels.

The changes in the imaginary part were more complex. Its value started high and dropped to a “valley” for low frequencies (approximately 3 GHz), which was followed by a “plateau” in the midrange of the measured frequencies (approximately 24 GHz) and a final drop for the highest measured frequencies (approximately 40 GHz). This irregularity decreased with the rise in the dehydration level, and the curve flattened for the highest dehydration levels (above 50%). We can also note that for the lowest frequencies, the imaginary part of the permittivity increased with the dehydration level.

#### 3.1.5. Visualising the Measurements for all Frequencies in 2D

**Figure 4:**
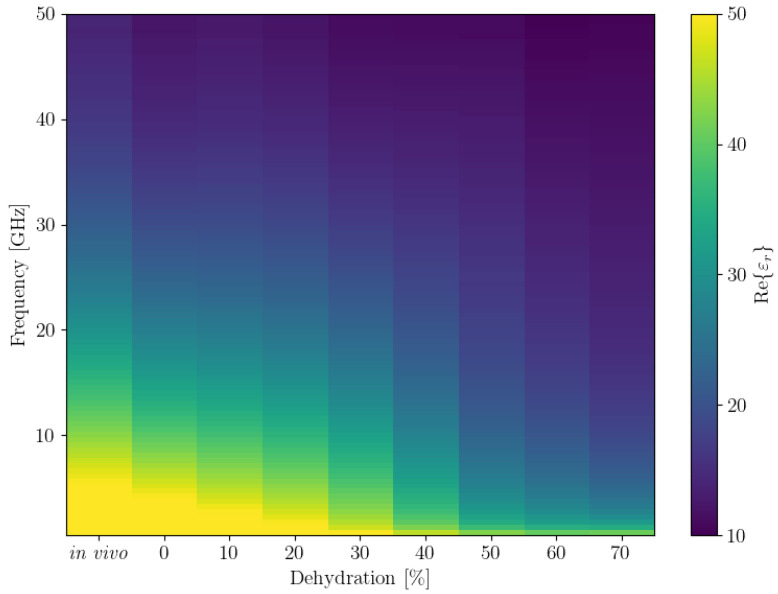

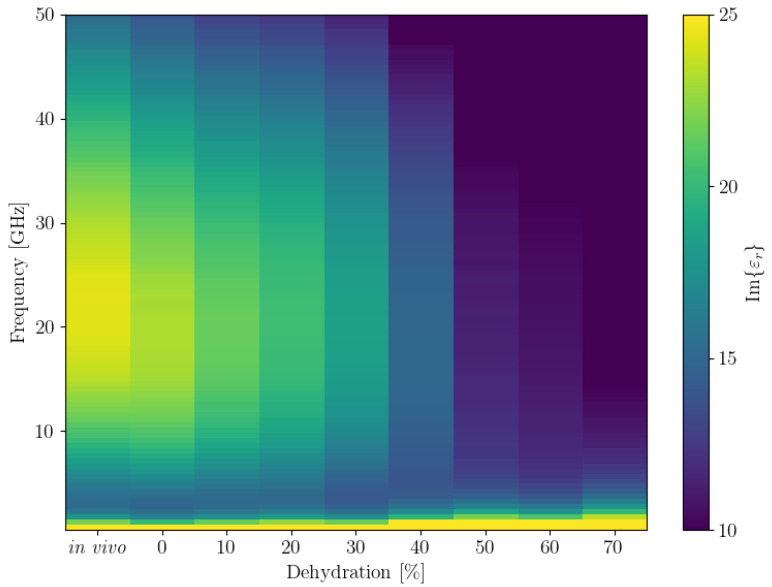
Binned and interpolated values of average real (**top**) and imaginary (**bottom**) parts of the permittivity ($\varepsilon_{r}^{\prime }$ and $\varepsilon_{r}^{\prime \prime }$, respectively) across all frequencies. Note that the colour bar limits were adjusted for better contrast, the actual ranges are 7–65 for Re($\varepsilon_{r}$) and 3–68 for Im($\varepsilon_{r}$).

We also plotted the average $\varepsilon_{r}^{\prime }$ and $\varepsilon_{r}^{\prime \prime }$ for all frequencies in a 2D plot, inspired by how spectrograms are represented. The results are shown in Figure 4. To obtain this plot, we first binned all the dehydration levels to bins that were multiples of 10. Since there were no data for the 40% dehydration bin, we then used linear interpolation to obtain the values for it. These plots show the trends we observed in the 3D plots of the data more clearly.

### 3.2. Regression Problem

#### 3.2.1. Restructuring with Average Nonuniform Samples

To formulate the task of estimating the in vivo permittivity from the measured ex vivo data, we chose to structure the data so that we grouped it by rat and averaged the measurements per dehydration level. Each average measurement is a sample with the target being the in vivo data for the rat measured. Since the targets are estimates of a continuous variable, it can be posed as a machine learning regression problem, in contrast to a classification problem in which the model is trained to estimate a target’s class. In our data re-organisation, we ignored the muscle identifier for the ex vivo measurements, as there was none for the in vivo ones.

Thus, we constructed a six-dimensional feature vector that comprised frequency, dehydration level, and the mean and standard deviation of both *ε*′ and *ε*″, extracted from the ex vivo data. The target was, in turn, two-dimensional comprising only the means of the *ε*′ and *ε*″ of the in vivo data. This restructuring of the data amounted to a total of 3634 observations.

Even though the data showed that the dynamics were influenced by the dehydration level, the measurement series made for different dehydration levels per rat cannot be directly used in most regression models because they are nonuniformly sampled. Namely, the permittivity was not measured at regular dehydration points across the different rats. Thus, we depended on the regression models to implicitly learn the influence of dehydration of the target values based on the set of training samples provided.

#### 3.2.2. Data Imputation

One way to deal with nonuniformly sampled data is through the use of data imputation, i.e., resampling the data at regular sample points using interpolated values from the original sampling of the data. To assess whether this approach was applicable to our data set, we took the mean data for rat 3 at a single frequency and used various interpolation and extrapolation methods to impute points over the whole range of dehydration levels. In particular, we used a spline interpolator with the following orders: 0 (zero), 1 (linear) and 2 (quadratic) as well as an interpolator imputing the value of the nearest known point (nearest).

**Figure 5:**
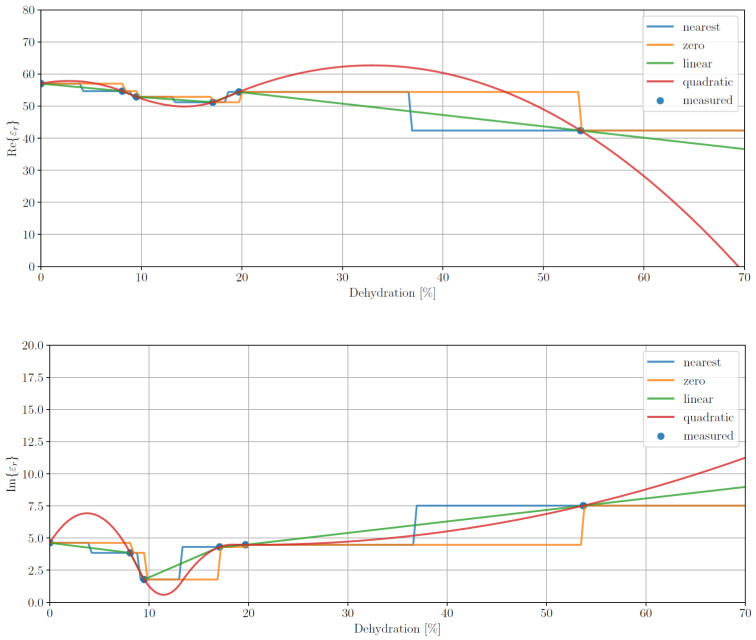
Interpolation permittivity results, average real (**top**) and imaginary (**bottom**) parts (*ε*′ and *ε*″, respectively), for data imputing for rat 3 at a frequency of 500 MHz for different interpolation methods: nearest neighbour (blue) and spline interpolation with orders of 0 (zero, orange), 1 (linear, green) and 2 (quadratic, red).

The results of this experiment are shown in Figure 5. The plots suggest that the usefulness of interpolation is limited by the access to only a handful of measurements to calculate the means. Unfortunately, due to the experimental nature of this study, we cannot explore imputing further. We do, however, publish these results so that other authors designing a measurement campaign can address this issue.

### 3.3. Chosen Regression Models

We based our analysis on the regression models implemented in the powerful machine learning package scikit-learn (Machine Learning in Python https://scikit-learn.org/, 13 October 2021). Indeed, scikit-learn provides a large variety of models from which to choose. From them, we chose to use models based on four different categories: linear regression, kernels, trees and tree ensembles, and neural networks. In total 11 models were selected, trained and evaluated.

#### 3.3.1. Linear Regression-Based Models

In our evaluation we incorporated:

- Linear regression—the ordinary least squares method evaluated for different degrees of the polynomial feature generator;
- Ridge regression—linear least squares with L2 regularisation of the weight parameters;
- Lasso regression—linear least squares with L1 regularisation of the weight parameters;
- Elastic net regression—linear least squares with combined L1 and L2 regularisation of the weight parameters.

#### 3.3.2. Kernel-Based Models

From the available kernel-based models we evaluated:

- Epsilon-support vector regression (SVR)—an extension of the support vector machines used for classification, the SVR supports various kernels from which we used the radial basis function (RBF) kernel;
- Kernel ridge regression (KRR)—combines ridge regression with the kernel trick to obtain a model identical in form to SVR but trained with a different loss function. Similarly, we again used the RBF kernel.

#### 3.3.3. Tree and Ensemble of Tree-Based Models

From the tree and ensemble of trees-based regressors we selected the:

- Decision tree regressor (regtree)—similarly to the decision tree classifier but outputs an estimate of the continuous target value from each leaf; splitting decisions are made in each node based on the best split among all the features;
- Random forest regressor (randforest)—an ensemble method based on averaging the output of a collection of decision tree regressors, each trained on a random subset of the data set; splitting is done with the best split among a random subset of all the features;
- Extremely randomised trees regressor (extratree)—an ensemble method based on averaging the output of a collection of decision tree regressors, each trained on a random subset of the data set; splitting is based on the best randomly chosen threshold for a random subset of features;
- Gradient boosted regression trees (gradboost)—an additive model made from a cascade of weak tree regressors, each trained on the previous one’s output.

#### 3.3.4. Neural Network-Based Models

From the neural network-based models, we used the multilayer perceptron (MLP) regressor. Since scikit-learn allows for building models with multiple hidden layers; we also trained and evaluated deep neural network (DNN)-based regressors.

### 3.4. Model Training and Evaluation

#### 3.4.1. Feature Augmentation

To increase the number of features available to the linear regression-based classifiers, we added polynomial feature combinations up to the 3rd degree. For example, a 2nd order polynomial feature generator that receives as input a two-dimensional feature vector (a, b), would output the six-dimensional feature vector (1, a, b, a^2^, ab, b^2^). This feature augmentation allows linear models to learn a polynomial data manifold.

#### 3.4.2. Data Normalisation

Since some regression models work better when the input features have similar scales, we included a standard scaler in the cross-validation pipeline. The standard scaler removes the mean and scales the features to unit variance as in:

(3) $$\hat{x}=\frac{x-\mu }{\sigma },\;$$

where *μ* is the mean value of feature *x*, and *σ* is its standard deviation.

An important caveat is that the data must be scaled using the mean and standard deviation of the training set exclusively. Doing otherwise would leak information from the validation or test sets into the model training.

#### 3.4.3. Hyperparameter Tuning

Random hyperparameter tuning was carried out for each of the selected regression models. For each hyperparameter, we defined a valid range of values and a random sampling function to sample from this range. Most hyperparameters were sampled using an exponential distribution, and some were sampled using a uniform distribution. Finally, some hyperparameter settings were limited to a list of values.

#### 3.4.4. Nested Cross-Validation Loop

Each of the models were trained and evaluated using a “leave one group out” nested cross-validation loop in which the data were grouped according to the rat identifier. In each iteration of the outer loop, one rat was selected to be the test rat. In the inner loop, each of the three remaining rats were taken out to be the validation rat in each iteration. Within each inner loop iteration, the model was trained with various hyperparameters and evaluated on the validation rat, and the hyperparameters that resulted in the best validation score were used to retrain the model for the three rats, i.e., including the validation rat. The newly trained model was then evaluated on the test rat. After the four rats were selected as test rats and the outer loop was completed, the average test score was calculated for the four rats.

The best results were selected based on the *R*^2^ coefficient of determination regression score measure defined as:

(4) $$R^{2}=1-\frac{\sum_{i=0}^{N-1}{\left(y_{i}-{\hat{y}}_{i}\right)}^{2}}{\sum_{i=0}^{N-1}{\left(y_{i}-\mu \right)}^{2}},$$

where *N* is the number of samples, *y* is the target value, *μ* is its average, and *y* is the target estimate output by the model. The best *R*^2^ score is a value close to 1, but the score can be negative. The advantage over using the mean square error (MSE), which is partly in the nominator in the above equation:

(5) $$\mathrm{MSE}=\frac{1}{N}-\sum_{i=0}^{N-1}{\left(y_{i}-{\hat{y}}_{i}\right)}^{2},\;$$

Is that a model that always predicts the mean target value, disregarding the input features, would have a *R*^2^ score of 0.

#### 3.4.5. Regression Models Results

The results obtained using the selected regression models on predicting the real and imaginary part of the in vivo permittivity, *ε*′ and *ε*″, based on its ex vivo measurements at various levels of dehydration, are shown in Table 2. The linear regression model was included to have a non-machine learning algorithm to predict in vivo dielectric properties. As expected, due to the pronounced non-linearity of the data, this algorithm performed the worst compared to the other machine learning algorithms. We can observe that most models do well on the set task, with the four best performing models being: extremely randomised trees, random forests, gradient boosted trees and regression trees, all scoring below 1.2 average MSE. From the remaining models, ridge regression is the next in line in terms of performance.

### 3.5. Most Important Features

**Figure 6:**
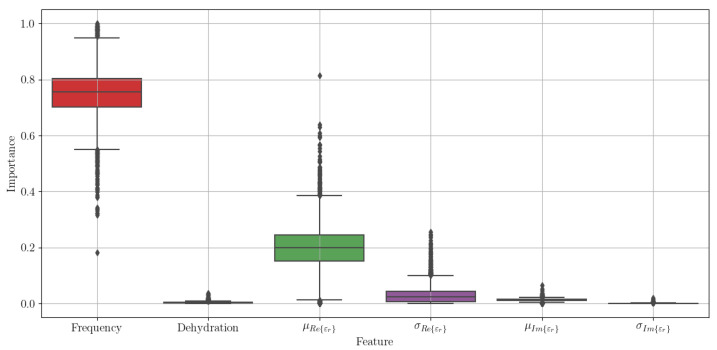
Feature importance across 1000 runs of the nested cross-validation loop for the extremely randomised trees regressor.

To estimate the most important features for estimating the in vivo target values, we took the best performing model—the extremely randomised trees—and repeated the nested cross-validation loop for 1000 iterations, aggregating the feature importance for each of the four models trained within one loop. Because of its stochastic nature, this process resulted with some 4000 trained models and as much feature importance values for each of the features. These are shown in the box plot in Figure 6. We can see that the two most important features are the frequency of the measured value and the mean of $\varepsilon_{r}^{\prime }$.

## 4. Conclusions

The presented results show that estimating the in vivo permittivity of muscle tissue in rats, based on measurements of its ex vivo permittivity is feasible, and current state-of-the-art regression models provide promising results. The models best suited for the task are ensemble tree-based methods, especially the extremely randomised trees regressor, which yielded the lowest MSE and highest *R*^2^ metrics. This study is an important first step towards the development of tools that can predict in vivo dielectric properties from widely available ex vivo dielectric properties.

In future work, we intend to include more metadata in our machine learning model to better estimate the in vivo dielectric properties of biological tissues as well as extending this study to other types of tissues other than rat muscle. Moreover, we will attempt to use a state-of-the-art model that can deal with nonuniformly sampled data, which is the recurrent neural network (RNN). In order to use this data with an RNN-based regressor, the data will have to be restructured in a dehydration series format.

## Figures and Tables

**Table 1:** Summary of the number of data points for each rat as measured in vivo, ex vivo (freshly excised) and at different dehydration levels.

Rat	In Vivo	Ex Vivo without Dehydration	Ex Vivo with Dehydration	Ex Vivo Dehydration Levels	Total
rat 3	808	808	4141	5	5757
rat 4	707	808	5959	10	7474
rat 5	808	808	3232	6	4848
rat 6	707	808	5555	9	7070
Total	3030	3232	18,887	30	25,149

**Table 2:** Average metrics obtained with the various regression models: linear regression (linear), ridge regression (ridge), lasso regression (lasso), elastic regression (elastic), support vector machines regressor (SVR), k-nearest neighbour regressor (KNNR), regression tree (regtree), random forest (randforest), extra trees (extratree), gradboost (gradient boost regression) and multilayer perceptron (MLP).

*Model*	*Number of Evaluated Hyperparameters*	*MSE* *Re(ε_r_)*	*MSE* *Im(ε_r_)*	*MSE* *Average*	*R^2^ Score* *Re(ε_r_)*	*R^2^ Score* *Im(ε_r_)*	*R^2^ Score* *Average*
linear	1000	3.03	3.52	3.28	0.982	0.667	0.825
ridge	1000	1.18	1.56	1.37	0.993	0.855	0.924
lasso	1000	1.33	1.59	1.46	0.992	0.855	0.924
elastic	1000	1.82	1.36	1.59	0.989	0.876	0.933
SVR	100	1.63	1.35	1.49	0.991	0.877	0.934
KRR	100	1.43	1.46	1.44	0.992	0.868	0.930
regtree	1000	1.25	0.99	1.12	0.993	0.913	0.953
randforest	100	1.11	0.98	1.05	0.994	0.913	0.954
*extratree*	*100*	*0.99*	*0.94*	*0.96*	*0.994*	*0.918*	*0.956*
gradboost	100	1.13	0.93	1.03	0.994	0.919	0.956
MLP	100	1.63	1.29	1.46	0.990	0.883	0.937
